# Impact of visual and auditory deprivation on speech perception: an EEG study

**DOI:** 10.1093/cercor/bhaf086

**Published:** 2025-04-29

**Authors:** Christine Turgeon, Vanessa Hadid, Paméla Trudeau-Fisette, Inga Knoth, Franco Lepore, Sarah Lippé, Lucie Ménard

**Affiliations:** Centre Interdisciplinaire de Recherche en Réadaptation et Intégration Sociale (Cirris), Département de réadaptation, Université Laval, 525 boulevard Wilfrid-Hamel, Québec, QC G1M 2S8, Canada; École de la Réadaptation, Université Laval, 1050 avenue de la Médecine, Québec, QC G1V 0A6, Canada; Laboratoire de phonétique de l’UQAM, Université du Québec à Montréal (UQAM), 405 rue Sainte-Catherine Est, Montréal, QC H2L 2C4, Canada; Centre for Research on Brain, Language and Music (CRBLM), 3640 rue de la Montagne, Montréal, QC H3G 2A8, Canada; CerebrUM, Département de psychologie, Université de Montréal, Pavillon Marie-Victorin, 90 avenue Vincent-d’Indy, Montréal, QC H2V 2S9, Canada; Department of Otolaryngology—Head and Neck Surgery, McGill University, McGill University Health Centre, 1001 Boulevard Décarie, Montréal, QC H4A 3J1, Canada; Laboratoire de phonétique de l’UQAM, Université du Québec à Montréal (UQAM), 405 rue Sainte-Catherine Est, Montréal, QC H2L 2C4, Canada; Centre for Research on Brain, Language and Music (CRBLM), 3640 rue de la Montagne, Montréal, QC H3G 2A8, Canada; CerebrUM, Département de psychologie, Université de Montréal, Pavillon Marie-Victorin, 90 avenue Vincent-d’Indy, Montréal, QC H2V 2S9, Canada; CerebrUM, Département de psychologie, Université de Montréal, Pavillon Marie-Victorin, 90 avenue Vincent-d’Indy, Montréal, QC H2V 2S9, Canada; CerebrUM, Département de psychologie, Université de Montréal, Pavillon Marie-Victorin, 90 avenue Vincent-d’Indy, Montréal, QC H2V 2S9, Canada; Laboratoire de phonétique de l’UQAM, Université du Québec à Montréal (UQAM), 405 rue Sainte-Catherine Est, Montréal, QC H2L 2C4, Canada; Centre for Research on Brain, Language and Music (CRBLM), 3640 rue de la Montagne, Montréal, QC H3G 2A8, Canada

**Keywords:** auditory evoked potentials, blindness, Cochlear implant, speech perception, speech production

## Abstract

This study investigated the impact of auditory and visual deprivation on speech processing by analyzing auditory evoked potentials (MMN, P3a, P2, N2b) in congenitally blind individuals, cochlear implant (CI) users, and normal-hearing controls. Using a passive oddball paradigm with /u/ as the standard stimulus and /i/ and /y/ as deviants, we recorded and analyzed auditory evoked potentials in fronto-central and centro-parietal regions. Blind participants exhibited significantly faster MMN and N2b latencies than controls and CI users, reflecting enhanced auditory temporal resolution due to cross-modal plasticity. CI users showed reduced P2 and N2b amplitudes, indicating challenges in early sensory processing and conflict monitoring, particularly for the /i/–/u/ contrast. Notably, blind participants had larger P3a amplitudes, emphasizing superior attentional engagement in response to deviant stimuli. Postlingually deafened CI users exhibited greater P3a amplitudes than prelingually deafened users, underscoring the impact of early auditory experiences on cortical responses. These findings demonstrate distinct effects of sensory deprivation on speech processing, with blind individuals showing compensatory neural mechanisms and CI users experiencing sensory and cognitive challenges. The results underscore the need for personalized rehabilitation strategies to enhance outcomes for populations with sensory deprivation and highlight the potential of cross-modal plasticity in auditory rehabilitation.

## Introduction

### The close links between speech production and speech perception and the importance of both hearing and vision in development

The important relationship between speech production and perception has been widely acknowledged. Over the years, researchers have demonstrated correlations between the quality of produced speech and the auditory discrimination skills in individuals with normal hearing (Perkell et al. 2004). Furthermore, speech and communication commonly involve both auditory and visual modalities (Rosenblum 2008). Extensive research in psychology, linguistics, and neurophysiology over the past 30 years has provided compelling evidence for the multimodal nature of speech processing. The contribution of visual cues to speech perception has been extensively studied (McGurk and MacDonald 1976; Robert-Ribes et al. 1998), establishing the prominent role of the visual modality in speech perception. Even within weeks of birth, hearing infants demonstrate awareness of the correspondence between lip movements and speech sounds, and they are influenced by conflicting auditory and visual information (eg Burnham and Dodd 2004). Individuals who struggle with acquiring intelligible speech production also exhibit poorer performance in speech-reading tasks (Woodhouse et al. 2009). Specifically, research has shown that children who consistently make atypical phonological errors also display a reduced ability to integrate auditory and visual speech information (Dodd et al. 2008).

### Auditory deprivation

In individuals with severe-profound hearing loss, such as those who rely on a cochlear implant (CI), it is possible to partially restore hearing to a certain extent. A cochlear implant functions by converting auditory signals into electrical impulses. Previous studies have established significant connections between the quality of speech production and auditory abilities, both in children (Turgeon et al. 2017) and in adults with CI (Turgeon et al. 2015; Turgeon et al. 2020). CI users often have a limited inventory of perceived sounds, and our research has demonstrated that speech sounds produced by them are less typical and less precise compared to individuals with normal hearing.

### Visual deprivation

Despite the primacy of hearing in the development of speech, vision also plays a critical role. In individuals with congenital blindness, it is known that the production of different phonemes differs acoustically from their sighted peers (McConachie 1990; Trudeau-Fisette et al. 2017). Based on acoustic measures, our group demonstrated a decrease in contrasts between pairs of vowels in blind subjects (Menard et al. 2009). Using articulatory measures, we also demonstrated that blind individuals take less advantage of their visible articulators but compensate with greater movement of the tongue (Menard et al. 2013). In fact, it was found that blind individuals used smaller differences in lip protrusion to manipulate the acoustical output of various phonemes but showed larger differences in tongue position and shape compared to sighted speakers (Menard et al. 2016; Turgeon et al. 2020).

In order to better understand how speech production is affected by auditory and visual deprivation, we recently looked at acoustic and articulatory measures of speech in three different groups of adults; CI users, blind, and a control group. We discovered that CI users produced smaller acoustic contrast between the front and back vowels, as well as atypical F1(first formant) and F2 (second formant) values (Turgeon et al. 2020). The impact of deafness on speech production was also observed on tongue position. In line with previous studies on the blind population, we also demonstrated that the impact of visual deprivation on speech production was reflected to the visible articulators. This group showed larger lip opening but smaller lip protrusion. In this study, we used behavioral tests to evaluate speech perception abilities. Our results showed no difference between the control group and the blind but a lower score for the CI, which was expected. In order to better understand the relation between speech production and perception, and to objectively evaluate speech perception, the present study was completed. We sought to better understand the influence of auditory and visual deprivation on speech perception and production by comparing spoken vowels perceived and produced by deaf adults who used CIs, congenitally blind adults, and adults with normal sight and hearing. We used electrophysiological (EEG) measures to assess speech perception abilities.

### Event-related potentials

Auditory evoked potentials have been used with considerable success to assess the integrity of the auditory system in pediatric and adult CI users (Kraus et al. 1993; Groenen et al. 1996; Gordon et al. 2005; Kelly et al. 2005; Micco et al. 1995) as well as in blind individuals (Liotti et al. 1998; Leclerc et al. 2000; Roder et al. 2000; Kujala et al. 2007). In audiology, behavioral methods such as tests of speech intelligibility are the primary tools used to investigate auditory performance. However, these tests are not always appropriate for the assessment of individuals with prelingual deafness or for those with prolonged auditory deprivation, as they generally present severe deficits in language and speech recognition. Electrophysiological methods are proposed as an alternative and complementary measure to behavioral tools.

### Mismatch negativity

The mismatch negativity (MMN) is of particular interest because it provides an objective and noninvasive electrophysiological measure of auditory discrimination. This is non-negligible given that auditory discrimination is essential for speech. The MMN is elicited by a series of rare and deviant stimuli embedded in a sequence of standard stimuli. It can be elicited by any discriminable occasional changes in a sound sequence irrespective of the direction of the subject’s attention or task (Rinne et al. 2000). It reflects pre-attentive stages of information processing and sensory analysis of auditory input and its encoding into the memory trace (Näätänen and Alho 1995). In adults with normal hearing, the MMN is typically characterized by a negativity occurring approximately 100 to 250 ms after the onset of the deviant stimulus and that is maximal over the fronto-central electrodes (for a review, see Näätänen 1990). The presence of this negative waveform indicates that the auditory system responds differentially to stimuli that have different acoustic profiles, suggesting a certain integrity of auditory processes. Considering the phonetic information present in speech sounds (for ex: /y/), this type of acoustic stimuli is more relevant to study speech perception than pure tones. In fact, when speech stimuli are used to elicit the MMN, it is thought to index preattentive speech discrimination (for a review of speech evoked potentials, see Martin et al. 2008). This measure is useful for the study of discrimination processes not only during normal development but also in clinical populations, such as people with CIs (Wable et al. 2000; Sharma and Dorman 2006; Turgeon et al. 2014) and people with blindness (Liotti et al. 1998; Kujala et al. 2007).

#### Sensory deprivation effects

In CI users, electrophysiological data of hearing perception differs widely among individuals. Based on their results on speech perception tests, CI users can be classified as good or poor users. MMN components in well-performing CI users can resemble those of normal hearing participants, while poor CI performers generally exhibit lower amplitudes and longer latencies in MMN responses (Kraus et al. 1993; Groenen et al. 1996; Zhang et al. 2011; Turgeon et al. 2014).

In blind individuals, auditory evoked potentials can differ from neurotypical ones depending on the tasks and stimuli used. MMN amplitudes are generally similar between blind and control participants when pure tones are used (Liotti et al. 1998; Kujala et al. 2007), but differences in latency have been observed, particularly with more complex stimuli (Feng et al. 2019).

### P300 component

The P300 component of the event-related brain potential (ERP) has been widely studied in fundamental and clinical studies and is thought to reflect attentional and memory processes, among others (Polich and Herbst 2000). The P300 component is a positive deflection in voltage elicited by the recognition of a rare stimulus (oddball paradigm) within a series of frequent stimuli. P300 latency generally varies between 250 and 350 ms; however, wider latency ranges have also been reported (Martin et al. 2008; Massa et al. 2011; Didone et al. 2016a; Didone et al. 2016b). It has been suggested that latency increases when triggered by stimuli of greater complexity, such as speech stimuli (Tampas et al. 2005; Massa et al. 2011; Alvarenga Kde et al. 2013). The P300 potentials are one of the most studied components in electrophysiology, and they have been used with different clinical populations, including blind and CI users (Yael Henkin et al. 2008; Henkin et al. 2009; Liotti et al. 1998; Wronka et al. 2008). A subtype of the P300, known as the P3a, is also observed in response to unexpected stimuli and is associated with automatic attentional orienting. The P3a is typically elicited by novel or deviant stimuli and is more frontally distributed, though it can be recorded from fronto-central regions as well. In blind individuals, auditory processing differences in both P300 and P3a have been observed, particularly in latency, reflecting compensatory mechanisms in attentional engagement during auditory tasks (Feng et al. 2019).

#### Sensory deprivation effects

Oviatt and Kileny demonstrated that CI users exhibited prolonged latency and reduced amplitude in the P300 component, particularly when signal contrast was reduced (Oviatt and Kileny 1991). In CI participants, both P300 and P3a responses to difficult-to-discriminate contrasts displayed longer latencies than responses to easier contrasts (Oviatt and Kileny 1991), and similar trends were found using speech stimuli (Micco et al. 1995; Kelly et al. 2005; Obuchi et al. 2012).

Studies suggest that the neural reorganization resulting from cross-modal plasticity enhances auditory processing capabilities in blind individuals as a compensation mechanism, particularly reflected in the P3a component (Roder et al. 2000; Merabet and Pascual-Leone 2010). Additionally, the reliance on auditory over visual information may facilitate rapid attentional switching to auditory deviances, leading to robust P3a responses (Kujala et al. 2007).

### P2 and N2b

While the MMN and P3a are typically observed in the fronto-central regions, we also investigated the P2 (150 to 250 ms) and N2b (200 to 400 ms), as the rare stimulus in the passive oddball paradigm used in this study elicited a distinct response, prompting further investigation of their links to speech perception. The P2 component is often associated with sensory processing and auditory discrimination (Simal et al. 2021), while the N2b is linked to cognitive control (Folstein and Van Petten 2007; Yu et al. 2015), and both components can show more posterior distributions, particularly under passive or less attentionally demanding conditions.

#### Sensory deprivation effects

In CI users, the P2 component, reflecting early sensory processing of auditory stimuli, is often preserved in CI users, but its amplitude and latency can vary depending on auditory performance and have been linked to speech perception outcomes (McGuire et al. 2021). While the role of the N2b component in CI users remains less well understood, some evidence suggests that differences in N2 responses may reflect variability in cognitive processing demands and speech perception abilities (Finke et al. 2016).

In blind individuals, the P2 component has been shown to be enhanced, indicating possible compensatory mechanisms for processing auditory information. Enhanced P2 amplitudes in blind participants are thought to reflect increased reliance on early auditory processing to compensate for the lack of visual input (Focker et al. 2015). Similarly, differences in N2b have been observed, with blind individuals exhibiting faster latencies and larger amplitudes compared to sighted controls, reflecting heightened cognitive processing of auditory stimuli (Kujala et al. 1995).

### Objectives

This study aims to provide a comprehensive understanding of how auditory and visual deprivation affect both speech perception and production by examining the perceptual characteristics of the vowels /i/, /y/, and /u/, chosen for their distinct auditory and visual properties (Turgeon et al. 2020). Using electrophysiological measures, we seek to objectively assess speech perception abilities in individuals with different sensory experiences. Specifically, we will analyze ERPs—including the P2, MMN, P3a, and N2b—across CI users, congenitally blind individuals, and normal-hearing controls to better understand the neural mechanisms underlying speech perception.

#### Specific objectives

MMN (150 to 250 ms) will be analyzed for signs of automatic auditory discrimination, with expectations of diminished amplitudes and increased latencies in CI users, indicative of impaired pre-attentive processing. P2 (150 to 250 ms), reflecting early sensory processing, is anticipated to vary in amplitude among the groups, affected by each group’s auditory sensitivity, particularly altered in CI users. N2b (200 to 400 ms), indicating conscious auditory discrimination, is expected to reveal faster or more robust responses in blind individuals due to compensatory neural mechanisms. P3a (250 to 500 ms), associated with the brain’s response to novel sounds, may show reduced amplitudes in CI users, reflecting deficits in involuntary attention switching.

## Materials and methods

### Participants

Participants were the same as in Turgeon et al. (2020). Twelve adults with one or two CIs (mean age = 41 years, SD = 11,7, min = 27, max = 67), 14 congenitally blind adults (mean age = 43 years, SD = 13,8, min = 25, max = 63), and 16 sight-normal hearing adults (mean age = 39 years, SD = 9,7, min = 28, max = 61) participated. All CI users suffered from severe, profound bilateral hearing loss before their surgery. They all used oral language as a primary mode of communication. The CI group presented detection thresholds with the implant that were above 40 dB HL (decibel hearing level) for all frequencies tested, corresponding to what is generally reported in the literature for CI users (Peterson et al. 2010). The clinical profiles of the CI users are presented in Table 1. Blind participants had a congenital, complete visual impairment, classified as class 3, 4, or 5 in the International Disease Classification of the World Health Organization (WHO). Table 2 presents the pertinent characteristics of the blind participants. All controls and CI users had perfect (20/20) vision or impaired vision corrected by lenses, resulting in near-perfect vision. Both blind and control participants had auditory detection thresholds below 25 dB HL at every frequency, which corresponds to normal hearing. Pure-tone detection thresholds were assessed using an adaptive method at 250, 500, 1000, 2000, and 4000 Hz with a supra-auricular earphone for the blind and normal-hearing group and in free-field for the CI group. Warbles tones were used for everyone. All participants had Canadian French as their first language and reported having no motor deficits, learning disability, or other known medical conditions. They all gave written, informed consent in accordance with the Board of Ethics of the University of Quebec in Montréal (UQAM) (No 4753) and of CHU Sainte-Justine (No 3986).

**Table 1:** Clinical profile of CI users.

**Sex**	**Age**	**Etiology of deafness**	**Age at deafness (years)**	**Age at implantation (years)**	**Side of the Implant**	**Pre-implant hearing thresholds** **^a^** **(MPT)**	**Number of electrodes**	**Type of cochlear implant**
F	35	Meningitis	3	8	R	>120/>120	9	Cochlear-Freedom
F	27	Unknown	8	10	R	>120/>120	6	Neurelec- Saphyr CX
F	41	Congenital	Birth	30	L	107/>120	16	Advances Bionic-Clarion
M	51	Congenital	Birth	1er:41, 2e: 49	Bil	95/93	14 Bil	Advances Bionic-Aida
M	50	Unknown	Birth	43	L	117/>117	20	Cochlear-Freedom
M	43	Hereditary	2	37	R	103/106	16	Advances Bionic-Clarion
F	29	Meningitis	2	22	L	110/91	22	Cochlear-Freedom
F	52	Unknown	10	1er:46, 2e: 52	Bil	118/107	16 Bil	Advances Bionic-Clarion
F	67	Hereditary	6	55	L	>105/>105	16	Advances Bionic-Clarion
F	44	Congenital	Birth	38	R	101/>120	22	Cochlear Freedom Nucleus
M	32	Congenital	Birth	1er:25. 2e; 32	Bil	107/107	24 Bil	Cochlear Freedom Nucleus
F	27	Congenital	Birth	16	R	>117/93	22	Cochlear-ESPrit 3G

^a^MPT = Mean of pure-tone (500, 1000, 2000 Hz). > no measurable response at the limit of the audiometer.

**Table 2:** Clinical profile of blind participants.

**Sex**	**Age**	**Etiology of blindness**	**Vision at birth**	**Current vision**
M	25	Microphthalmia—congenital	Total blindness	U^a^
				(total blindness)
F	32	Retinoblastoma—congenital	Total blindness	U
				(total blindness)
M	23	Detachment of the retina—1 week	Total blindness	U
				(total blindness)
M	27	Retinoblastoma—9 months	U	U
				(total blindness)
F	59	Microphthalmia—congenital	Total blindness	U
				(total blindness)
M	52	Optic atrophy—congenital	U	U
				(total blindness)
F	60	Detachment of the retina	U	U
				(total blindness)
M	46	Detachment of the retina	U	U
				(total blindness)
M	50	Unknown	Total blindness	R.E.^b^ = 20/400
				L.E.^c^ = 20/400
F	50	Congenital cataract	U	R.E. = 0
				L.E. = 6/1260
M	39	Unknown	U	U
				(total blindness)
M	63	Congenital cataract	Total blindness	U
				(total blindness)
M	28	Leber’s congenital amaurosis—congenital	U	U
				(total blindness)
M	45	Retinitis pigmentosa	Total blindness	U
				(total blindness)

^a^Undetermined.

^b^Right eye.

^c^Left eye.

### Apparatus

EEG recording took place in a dark soundproof chamber in the Sainte-Justine Hospital. Stimuli were presented via a Dell optiplex 790 PC using E-Prime 2.0.8.90 (Psychology Software Tools Inc. Pittsburgh, PA, USA). Two speakers (BX5a, M-Audio, Canada) were placed laterally at a 30-cm distance from the subject’s ears.

### Stimuli

The stimuli consisted of three synthesized 5-formant vowels of French (/i/, /y/, /u/). Formants refer to the broad spectral maximum that results from an acoustic resonance of the human vocal tract. Each vowel can be described in terms of distinct formants, the first 3 being the most distinctive ones. The vowels we used were selected because they are characterized by different auditory and visual perceptual saliency. For the /i/ vs. /y/ pair, only the protrusion of the lips varies (visible articulators), the front position of the tongue (nonvisible articulator) remaining unchanged. Therefore, they differ in terms of F2 and F3. For the /y/ vs. /u/ pair, only the position of the tongue varies (front for /y/ vs. back for /u/), while the position of the lips remains unchanged. This articulatory movement of the tongue causes an important variation of F2 values. Finally, the pair /i/ vs. /u/ implies a more complex articulatory contrast where both the visible and nonvisible articulators are involved (front tongue position and unrounded lips for /i/ vs. back tongue position and rounded lips for /u/). Again, those articulatory differences are reflected in F2 and F3 values. As all three vowels are produced with an almost closed oral cavity (also called high vowels), they all show similar F1 values. These vowels were a subset of those that served as stimuli in a previous speech discrimination experiment (Menard et al. 2009). They were created with the Variable Linear Articulatory Model. Formant and bandwidth values are presented in Table 3. All stimuli were 225 ms long. Prior to the testing, each CI participant was asked to adjust their implant processors at their usual setting so they could hear the stimuli at a comfortable loudness level. Subjectively reported by the participant, all stimuli were heard with the same comfortable loudness.

**Table 3:** Formant (*F*_i_) and bandwidth (*B*_i_) values, in hertz, of end-point stimuli /i/, /y/, and /u/ synthesized for the perceptual experiment.

**Vowel**	**F1**	**F2**	**F3**	**F4**	**F5**	**B1**	**B2**	**B3**	**B4**	**B5**
**/i/**	236	2,062	3,372	3,466	5,000	78	13	61	154	154
**/y/**	236	1,757	2,062	3,294	5,000	88	40	19	19	19
**/u/**	236	705	2,062	3,294	5,000	88	40	19	19	19

### Electrophysiological recording

#### The passive task

Stimuli were presented using a two-deviant oddball paradigm in which /u/ was the standard (probability of occurrence = 84%) and /y/ and /i/ were the deviants (probability of occurrence = 8% each). The interstimulus interval was 700 ms. Stimuli were presented in pseudo-random order with at least three standard stimuli presented before the presentation of a deviant stimulus. The task contained five blocks of 330 standard and 30 deviant stimuli each. Altogether, 1,650 standards and 150 of each deviant were presented. During the recording, participants were asked to read a book. Knowing that Braille reading requires light finger movements, we asked the CI users and control participants to read by doing the same kind of movement in order to control for the motor aspect of the task.

#### Electroencephalography recordings

Electroencephalography (EEG) was measured using a 128-channel dense array electrode system (Electrical Geodesics System Inc., Eugene, OR, USA). EEG data were digitalized by a G4 Macintosh computer using NetStation Software (Version 4.5.4). Participants temporarily removed their CI during the installation of the electrode net to avoid device damage. Electrode impedance was kept below 40 kΩ before baseline recording, which is the standard for high-input-impedance amplifiers (Tucker 1993). An additional impedance measurement was performed in the middle of the recording session to assure that impedance remained below 40 kΩ.

#### Data analysis

A problematic factor well known with the EEG signals measured in CI populations is the artifacts induced by the implant device. These artifacts are perfectly time-locked to the acoustic stimulus and can lead to larger amplitudes than the one induced by the stimulus (Gilley et al. 2006; Debener et al. 2008). To avoid an overestimation of the cortical responses evoked by the acoustic stimuli, it is imperative to detect these artifacts and remove them. Several techniques are proposed, and independent component analysis (ICA) has been suggested as one of the most effective techniques to remove EEG artifacts (Gilley et al. 2006). In fact, when used with a large number of recording electrodes, ICA greatly minimizes the implant artifacts (Gilley et al. 2006). ICA decomposition of the EEG signal provides spatially fixed and temporally independent components (Debener et al. 2005). We used ICA analysis for all groups to remove CI device–induced and ocular artifacts in the EEG signal. This method is well described in Gilley et al. (2006).

All analyses were performed with Brain Vision Analyzer version 2.1 (Brain Products GmbH, Munich, Germany). Thirty electrodes containing muscular artifacts, around the neck and face, were removed for all participants. First, high- and low-pass filters were set at 0.1 and 30 Hz (24 dB/octave). Data were re-referenced to the average reference for all groups. ICA, as implemented in Brain Vision Analyzer version 2.1, was then applied to all data, for all groups. Following that, components coming from the CI device and/or the eye movements were removed from the data. Component activations were treated as CI artifacts if they met the following criteria, as described and applied in Gilley et al. (2006) and in Turgeon et al. 2020: (i) the onset/offset of activity occurred at the onset/offset of the auditory stimulus; (ii) the duration of the activity was constant throughout the duration of the auditory stimulus; and (iii) scalp projections of the activity revealed a centroid on the side of the implant device. The EEG data were segmented into epochs with each epoch beginning 100 ms before stimulus onset and ending 600 ms after stimulus onset. A local DC trend correction and a baseline correction within the prestimulus interval were applied to the segments. A semi-automatic artifact rejection was then carried out; the algorithm marked segments containing activity exceeding ±100 μV that were removed during a subsequent manual inspection in addition to any other artefactual sporadic activity. A grand average for all stimuli was computed for each participant.

#### ERP component analysis

The study focused on the quantification of event-related potentials (ERPs) for both deviant and standard stimuli, as well as their difference waves (deviant minus standard). Specifically, the analysis targeted the MMN and P3a components in the fronto-central region and the P2 and N2b components in the centro-parietal region. The MMN was computed by subtracting each participant’s average response to the standard stimulus from their response to the deviant stimulus. Electrodes around FCz and Cz were used to examine the MMN, as this component typically reaches its maximum amplitude in the fronto-central region (Ilvonen et al. 2004; Näätänen et al. 2004; Duncan et al. 2009). To improve the signal-to-noise ratio, EEG activity was pooled across five fronto-central electrodes and five centro-parietal electrodes. Approximate anatomical labels were assigned based on spherical coordinates (theta and phi) from the EGI 128-channel system, using the 10–10 international system from a 64-channel layout as reference. The fronto-central electrodes were centered on FCz and extended slightly below and laterally—leftward and rightward —along the fronto-central midline. The centro-parietal electrodes were centered on Pz, extending slightly above and below on the left and right hemispheres. Component-specific time windows were defined based on typical ERP latencies: 150 to 250 ms for MMN and P2, 250 to 500 ms for P3a, and 200 to 400 ms for N2b. Within these windows, peak amplitude (in microvolts, μV) and peak latency (in milliseconds, ms) were extracted for each participant. Peak amplitude was defined as the maximum (for positive components) or minimum (for negative components) voltage within the specified time window. Peak latency corresponded to the time point at which this maximum or minimum amplitude occurred. All EEG epochs were baseline-corrected using the mean voltage of the prestimulus interval (−100 to 0 ms).

### Statistical analysis

Statistical analyses focused on comparing ERP amplitudes and latencies across the control, blind, and CI groups based on the specific auditory contrasts /i/–/u/ and /y/–/u/. Analysis of variance (ANOVA) was utilized to explore the main effects of Group and Condition, as well as their interaction, on both amplitude and latency measurements. For each component and pooling (fronto-central for MMN and P3a; centro-parietal for P2 and N2b), ANOVAs were conducted to assess the differential responses elicited by phonetic variations in auditory stimuli. When significant effects or interactions were detected, post-hoc pairwise comparisons were performed using Tukey’s Honestly Significant Difference method to elucidate the specific group and condition differences.

### Investigating CI variability: group differences and correlation analyses

To account for the variability among CI users, two groups were formed based on their age at onset of deafness: at or before 3 years old (≤3 years) and after 3 years old (>3 years). This division was informed by developmental linguistics, as the age of 3 years generally marks the transition from a prelinguistic to a postlinguistic phase, where foundational language skills are largely established (Watt et al. 2006).

Independent-samples *t*-tests were conducted for each ERP component (MMN and P3a in the fronto-central region; P2 and N2b in the centro-parietal region) and for each measure (amplitude and latency) to compare neural responses between the two groups. These analyses were initially conducted separately for the /i/–/u/ and /y/–/u/ auditory contrasts. However, as both contrasts yielded similar results, the data were combined for simplicity. The significance threshold was set at *P* < 0.05.

To further investigate the observed variability in neural responses, correlation analyses were performed to examine the relationships between the amplitudes and latencies and age at deafness and age at implantation. Pearson’s correlation coefficient was calculated, and linear regression was used to model these relationships. Age at deafness and age at implantation were treated as independent variables, and the amplitudes and latencies of each component were treated as the dependent variable. These analyses were also performed on the combined data from the /i/–/u/ and /y/–/u/ contrasts. The regression models provided *R*^2^ values to quantify the proportion of variance explained by each factor, as well as adjusted *R*^2^ values to account for the sample size and number of predictors. The statistical significance of the correlations was determined by the associated *P*-values, with a threshold of *P* < 0.05.

## Results

### ERP differences between controls, blinds, and CI users measured across time

Our EEG analysis revealed distinct patterns of ERPs within the fronto-central and centro-parietal regions across all participant groups. In the fronto-central region, we observed a consistent negativity for both the /i/ and /y/ deviant stimuli detectable in the 150- to 250-ms poststimulus interval, suggesting MMN that can be reliably induced without the necessity for directed attention across control, blind, and CI groups. A significant cluster around the MMN was specifically observed in the blind group, where the /i/ stimulus induced significantly greater negativity compared to the standard stimulus /u/ (*P* = 0.23). Although this negativity was observed in all groups, it was less prominent in the control and CI user groups.

**Fig. 1:**
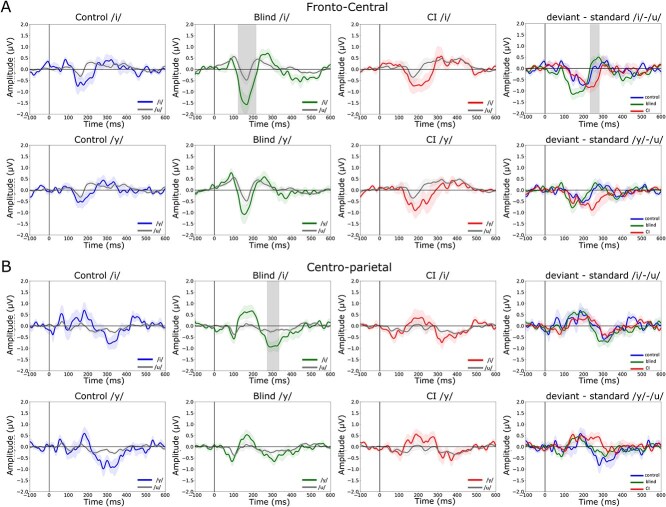
Comparison of EEG activity between control, blind, and CI groups for the deviant—standard contrasts in fronto-central A) and centro-parietal B) regions. Amplitude (μV) is plotted over time (ms), and shaded areas around the waveforms represent the standard error of the mean (SEM). A) Fronto-central activity for the /i/–/u/ contrast in the first row, and /y/–/u/ contrast in the second row. B) Centro-parietal activity for the same contrasts: /i/–/u/ in the first row and /y/–/u/ in the second row. In the first three columns, waveforms represent responses to the standard condition (/u/) and the deviant condition (/i/ or /y/) for each group (control, blind, CI). The fourth column shows the difference waveforms (deviant minus standard) for each group. Statistical significance: Clusters where the differences between groups were statistically significant (*P* < 0.05) are highlighted in gray within each plot. These significant clusters indicate time intervals where EEG amplitudes differed significantly between the deviant and standard in the first three columns and between groups in the last column. We identified specific time intervals of interest: The MMN (150 to 250 ms) and P3a (250 to 500 ms) components for the fronto-central region and the P2 (150 to 250 ms) and N2b (200 to 400 ms) components for the centro-parietal region.

Subsequently, a notable P3a component emerged in the 250- to 500-ms window across all groups for both the deviant stimuli /i/ and /y/. Comparative analysis between the control, blind, and CI groups revealed significant differences in response to deviant and standard stimuli, with the blind group exhibiting the highest P3a amplitude. This effect was specifically noted between 222 and 315 ms poststimulus onset in fronto-central regions during the comparison of /i/ and /u/ (*P* = 0.015) (Fig. 1A).

In the centro-parietal region, significant responses associated with the P2 (150 to 250 ms) and N2b (200 to 400 ms) components were detected in response to the deviant stimuli. However, the blind group again showed the most substantial differences from 272 to 333 ms (*P* = 0.04) (Fig. 1B). Differences between the /y/ and the /u/ were less prominent in both centro-parietal and fronto-central regions.

### Differential responses in centro-parietal region between the control and CI groups

Subsequently, we focused on quantifying the amplitude and latency of the key ERP components, MMN and P3a in the fronto-central regions and P2 and N2b in the centro-parietal regions. By examining these components, we aimed to capture the differences in how distinct sensory deprivation alters or enhances the processing of deviant auditory stimuli across the conditions /i/–/u/ and/y/–/u.

ANOVA pairwise comparisons revealed significant differences in the amplitudes of ERP components between control and CI groups, particularly in the centro-parietal region. These differences were observed differently between both deviant–standard contrasts (/i/–/u/ and /y/–/u/).

**Fig. 2:**
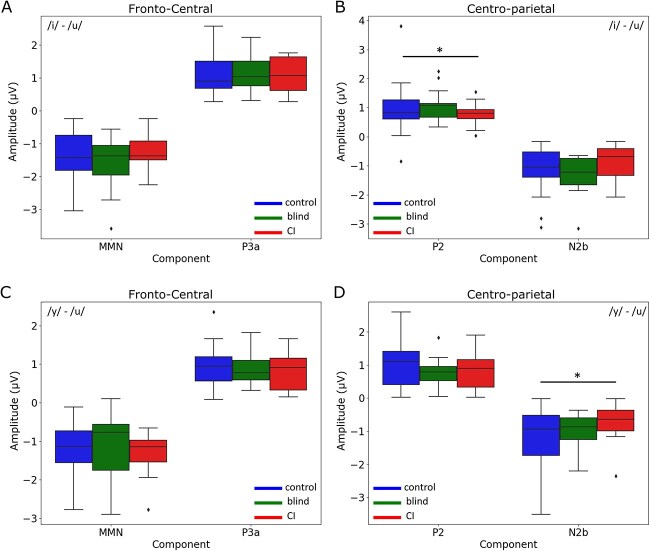
Comparison of amplitude differences between groups (control, blind, CI) for fronto-central and centro-parietal regions in the /i/–/u/ and /y/–/u/ conditions. A) Fronto-central region amplitude for the /i/–/u/ condition, highlighting MMN (150 to 250 ms) and P3a (250 to 500 ms). B) centro-parietal region amplitude for the/i/–/u/ condition, showing P2 (150 to 250 ms) and N2b (200 to 400 ms). C) Fronto-central region amplitude for the /y/–/u/ condition, illustrating MMN and P3a. D) centro-parietal region amplitude for the /y/–/u/ condition, depicting P2 and N2b. Boxplots represent the distribution of peak amplitudes (μV) within each component’s time window for each group, with the mean and SEM. Statistically significant differences between groups (*P* < 0.05) are indicated by asterisks and horizontal bars.

In fact, significant differences were detected in the amplitudes of both P2 and N2b components when comparing responses to the /i/–/u/ contrast. The P2 component showed a decreased amplitude in CI users compared to controls, with a mean difference of 0.2314 (*P* = 0.0479, 95% CI [0.0021, 0.4607]) (Fig. 2B).

Significantly, for the /y/–/u/ contrast, the N2b component, showed a pronounced decrease in amplitude in CI users compared to controls. This means that in controls, the N2b component was more negative, with a mean amplitude difference of −0.2733 (*P* = 0.0363, 95% CI [−0.5327, −0.0138]) (Fig. 2D).

No significant amplitude differences were detected for the MMN and P3a components across groups within the fronto-central regions for either contrast. However, our analysis revealed differences in the latency of these responses as described below.

### Faster ERP responses for blind individuals

The analysis of latency differences for the MMN component in the fronto-central region revealed significant variations between groups when responding to both the /i/–/u/ and /y/–/u/ contrasts, reflecting differing neural timing in processing these auditory deviations.

**Fig. 3:**
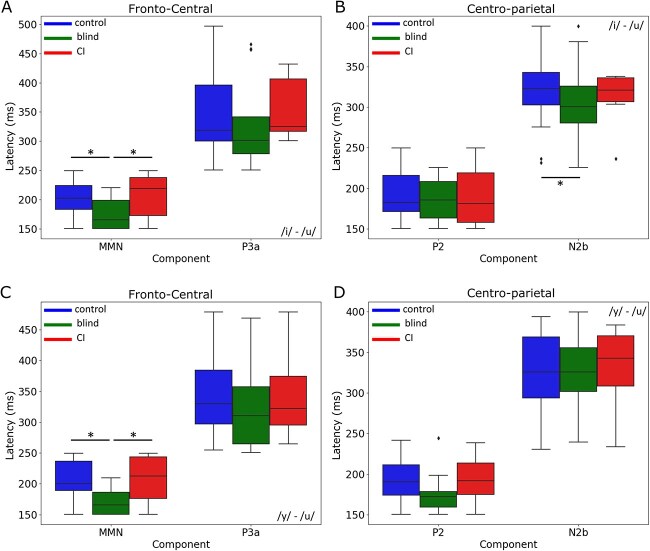
Comparison of latency differences between groups (control, blind, CI) for fronto-central and centro-parietal regions in the /i/–/u/ and /y/–/u/ conditions. A) Fronto-central region latency for the /i/–/u/ condition, highlighting MMN (150 to 250 ms) and P3a (250 to 500 ms). B) centro-parietal region latency for the /i/–/u/ condition, showing P2 (150 to 250 ms) and N2b (200 to 400 ms). C) Fronto-central region latency for the /y/–/u/ condition, illustrating MMN and P3a. D) centro-parietal region latency for the /y/–/u/ condition, depicting P2 and N2b. Boxplots represent the distribution of peak latencies (ms) within each component’s time window for each group, with the mean and SEM. Statistically significant differences between groups (*P* < 0.05) are indicated by asterisks and horizontal bars.

- **For the contrast /i/**–**/u/—**The ANOVA revealed significant differences in latency with an *F*-value of 7.6119 [df = 1, PR(>*F*) = 0.0093] where blind participants demonstrated a faster MMN response compared to controls by an average of 29.8386 ms (p-adj = 0.0093, 95% CI [7.8596, 51.8176]) and to CI users, with an *F*-value of 6.2665 (df = 1, PR(>*F*) = 0.0206) with an average advantage of 33.8792 ms (p-adj = 0.0206, 95% CI [5.7341, 62.0243]) (Fig. 3A). Blind participants exhibited a notably faster N2b response than controls, with a mean latency difference of 53.0591 ms (*P* = 0.0168, 95% CI [10.1685, 95.9496]) (Fig. 3B).
- **For the contrast** /**y/**–**/u/—**Significant latency differences were further highlighted with an *F*-value of 14.2002 [df = 1, PR(>*F*) = 0.0006] where blind participants had faster MMN responses by an average of 36.7582 ms (p-adj = 0.0006, 95% CI [16.9346, 56.5818]) compared to controls and of an *F*-value of 7.4315 (df = 1, PR(>*F*) = 0.0127) compared to CI users by an average of 35.7742 ms (p-adj = 0.0127, 95% CI [8.4835, 63.0649]) (Fig. 3C).

### Effect of age at deafness on ERPs in the CI group

**Fig. 4:**
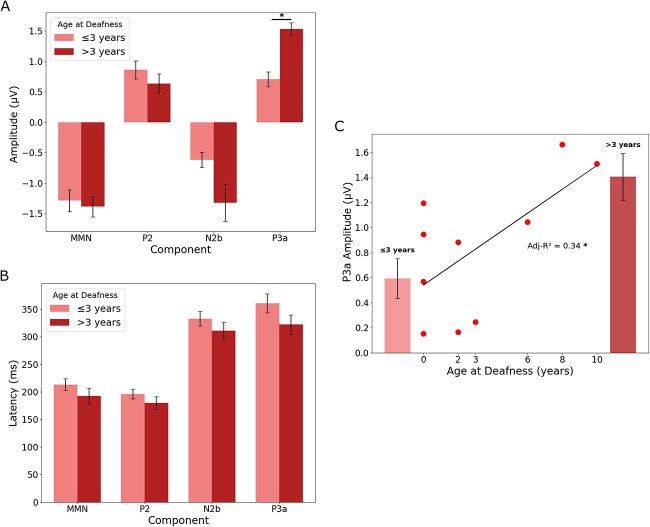
Impact of age at onset of deafness on ERP components in CI users. A) ERP component amplitudes: mean peak amplitudes (μV) of four ERP components—MMN and P3a (from the fronto-central region) and P2 and N2b (from the centro-parietal region)—elicited using the deviant–standard paradigm and averaged across /i/–/u/ and /y/–/u/ contrasts. CI users are grouped by age at onset of deafness: ≤3 years and >3 years. Error bars indicate SEM. Significant group differences are observed for the P3a component. B) ERP component latencies: mean peak latencies (ms) for the same components and groups, with identical color coding and error bar conventions as in (A). (C) Correlation between age at onset of deafness and P3a amplitude: relationship between age at onset and mean P3a peak amplitude (adjusted *R*^2^ = 0.34, *P* < 0.05). Group means are shown with SEM (≤3 years; >3 years).

We investigated the impact of age at onset of deafness on the electrophysiological signatures of CI users, focusing on four key ERP components: MMN, P2, P3a, and N2b. CI users were dichotomized into two groups based on the age at onset of deafness at or before 3 years (≤3 years; *n* = 6) and after 3 years (>3 years; *n* = 3). The patterns observed were consistent across different phonemic contrasts (/i/–/u/ and /y/–/u/), which is why visualization was made on the combined values, but below, we describe them separately (Fig. 4).

#### M‌MN amplitude

For the/i/–/u/ contrast, no significant differences in amplitude were observed between the ≤3 years and >3 years groups, with MMN amplitudes of −1.18 and −1.60 μV, respectively (*P* = 0.331). Similarly, for the /y/–/u/ contrast, MMN amplitudes were −1.40 μV for the ≤ 3 years group and −1.17 μV for the >3 years group (*P* = 0.633), with latencies of 214 and 192 ms, respectively (*P* = 0.478) (Fig. 4A).

#### P2 amplitude

No significant differences were observed for P2 amplitudes in both contrasts. For/i/–/u/, amplitudes were 0.80 μV for the ≤3 years group and 0.73 μV for the >3 years group (*P* = 0.836), and, for /y/–/u/, amplitudes were 0.92 and 0.54 μV, respectively (*P* = 0.391) (Fig. 4A).

#### N2b amplitude

Although no statistically significant differences were found for the N2b component amplitudes in both phonemic contrasts, a notable pattern emerged. For the /i/–/u/ contrast, we observed an increase in N2b amplitude for the group that acquired deafness postlinguistically (>3 years), with amplitudes of −0.70 μV for the ≤3 years group compared to −1.33 μV for the >3 years group. Similarly, for the /y/–/u/ contrast, the amplitudes were −0.54 μV for the ≤3 years group and −1.32 μV for the >3 years group (Fig. 4A).

#### P3a amplitude

Significant differences were observed in the P3a amplitudes for the /i/–/u/ contrast, where the >3 years group exhibited higher amplitudes (1.66 μV) compared to the ≤3 years group (0.82 μV), with a significant *P*-value of 0.024. The /y/–/u/ contrast also showed significant differences in P3a amplitudes, with 0.59 μV for the ≤3 years group and 1.40 μV for the >3 years group (*P* = 0.019) (Fig. 4A).

#### Latencies

Furthermore, latencies across all ERP components tended to be faster for the postlinguistically deafened group while not being significant (Fig. 4B).

#### Correlation

A significant correlation was found between age at deafness and P3a amplitude, elicited using the deviant–standard paradigm and averaged across /i/–/u/ and /y/–/u/ phonemic contrasts, characterized by an adjusted *R*^2^ of 0.34 (*P* < 0.05) (Fig. 4C).

## Discussion

This study aimed to investigate how auditory and visual deprivation influences speech perception and processing by analyzing ERPs in CI users, congenitally blind individuals, and normal-hearing controls within a single experimental paradigm. We focused on key ERP components, MMN, P3a, P2, and N2b, using deviant vowel stimuli contrasts (/i/–/u/ and /y/–/u/) to understand differences in auditory processing across groups.

### Differential processing of automatic detection across groups

Our analysis of the MMN (150 to 250 ms poststimulus) in the fronto-central region revealed a reliable negativity for both /i/ and /y/ deviant stimuli across all groups, indicating successful auditory discrimination without directed attention. This supports previous findings that the MMN can serve as a robust measure of auditory processing across different populations (Näätänen and Alho 1995; Näätänen et al. 2007). However, no significant differences in MMN amplitude were found between groups. This aligns with studies showing that well-performing CI users can display MMN responses comparable to those of normal-hearing individuals, although poorer CI performers may show attenuated responses (Groenen et al. 1996; Zhang et al. 2011; Groenen et al. 2001). It is also likely that the lack of significance in MMN amplitude differences may be attributed to the small sample size.

### Enhanced auditory processing in blind individuals: evidence from ERP components

Blind individuals demonstrate significantly enhanced auditory processing, as indicated by faster and more robust ERP responses. In particular, blind participants exhibited higher P3a amplitudes than CI users for the /i/–/u/ contrast, suggesting that blind individuals may possess enhanced cognitive mechanisms for processing phonetic variations. This supports well-established cross-modal plasticity frameworks (Bavelier and Neville 2002; Merabet and Pascual-Leone 2010; Fieger et al. 2006), where deprivation of one sensory modality leads to cortical reorganization in regions typically dedicated to the deprived modality. Specifically, the occipital cortex in blind individuals has been shown to be recruited for auditory attention tasks (Voss and Zatorre 2012; Collignon et al. 2009) and phoneme discrimination (Fine and Park 2018), likely underpinning both faster MMN latencies and enhanced P3a amplitudes observed in this group.

Blind participants also consistently exhibited faster MMN and N2b latencies compared to both controls and CI users. For the /i/–/u/ contrast, blind participants demonstrated significantly faster MMN latencies than controls (*P* = 0.0093) and CI users (*P* = 0.0206), with average differences of 29.8 and 33.9 ms, respectively (Fig. 3A). This pattern was also observed in the N2b component, where blind participants exhibited a mean latency advantage of 53.1 ms over controls (*P* = 0.0168) (Fig. 3B). For the /y/–/u/ contrast, similar latency advantages were observed for the blind group, with significantly faster MMN responses compared to both controls (*P* = 0.0006) and CI users (*P* = 0.0127). Taken together, these results support the hypothesis that blind individuals develop compensatory mechanisms that enhance the efficiency of auditory processing, likely due to the neural plasticity associated with early sensory deprivation (Feng et al. 2019).

From a cognitive adaptation perspective, blind individuals invest additional neural resources into auditory processing due to their reliance on auditory input for spatial navigation and communication (Kujala et al. 2007; Collignon et al. 2011). This increased reliance on auditory cues enhances their ability to detect and orient to novel auditory stimuli, as reflected in their stronger and faster ERP responses. These adaptations highlight the brain’s remarkable plasticity in reorganizing sensory pathways to compensate for deprivation (Kujala et al. 2000).

It is also important to distinguish between lifelong neural adaptations and the possible effects of specific sensory or cognitive training. While our findings suggest a robust compensatory mechanism driven by cross-modal plasticity, these enhancements may also be influenced by systematic training in auditory tasks, which could selectively sharpen auditory discrimination skills. Further studies could elucidate the relative contributions of innate neural reorganization versus targeted training regimens in shaping these auditory capabilities.

### Challenges in auditory discrimination for CI users: insights from ERP responses

CI users showed altered ERP amplitudes for P2 and N2b components, which reflect sensory processing and cognitive control, respectively (Stapells 2002). The P2 component, typically associated with early sensory processing, was significantly reduced in CI users compared to controls for the /i/–/u/ contrast. This suggests that normal-hearing individuals demonstrate superior early-stage sensory processing for contrasts involving both place of articulation and lip rounding.

For the N2b component, which could reflect more demanding phonetic processing, CI users exhibited a significant reduction in amplitude compared to controls for the /y/–/u/ contrast (*P* = 0.0363). This finding mirrors prior research showing that CI users often struggle with phonetic discrimination tasks, particularly when contrasts involve subtle articulatory differences (Beynon et al. 2005; McMurray and Apfelbaum 2019). The diminished N2b response highlights deficits in higher-order auditory processing, suggesting that CI users may require more effort to resolve complex auditory conflicts.

The differences in the deviant stimuli contrasts, specifically between /i/ and /u/, have led to more pronounced and reliable results compared to the /y/–/u/ contrast. The /i/–/u/ contrast, involving both place of articulation (front vs. back tongue position) and lip rounding (unrounded vs. rounded), presents a relatively distinct and easier challenge in terms of phonetic discrimination. These vowels differ significantly in both articulatory and acoustic features, particularly in the second formant (F2), making the contrast more pronounced (Turgeon et al. 2020). In comparison, the /y/–/u/ contrast involves only a change in tongue position, with consistent lip rounding, which presents a subtler difference and is therefore more challenging to detect, particularly for CI users, who often struggle with more nuanced articulatory contrasts (Turgeon et al. 2020). The distinct articulatory and acoustic features between /i/ and /u/ likely facilitate greater phonetic discrimination (Sanju and Kumar 2016).

These observations are consistent with broader auditory perception research suggesting that the degree of phonetic contrast affects the salience of auditory stimuli, which, in turn, influences neural processing efficacy (Iverson and Kuhl 1995; Näätänen et al. 1997). While our results do not directly link specific phonetic properties to ERP outcomes, they reinforce the importance of phonetic contrast in shaping auditory processing, especially in sensory-deprived populations. Future research could explicitly explore these links to better understand how different degrees of phonetic contrast impact ERP responses across varied auditory conditions.

### Impact of pre- and postlingual deafness on CI users

Stratifying our CI group by age at onset of deafness (≤3 years vs. >3 years) provides important insights into how early auditory experience shapes cortical processing following implantation. The early ERP components (MMN and P2), often linked to initial auditory discrimination and sensory registration, showed minimal amplitude differences relative to deafness onset. This suggests that foundational auditory processes may sufficiently be compensated for in both pre- and postlingually deafened individuals under our testing conditions (Giraud and Lee 2007; Kral and O’Donoghue 2010). However, faster latencies for all ERP components in the postlingual group highlight a timing advantage likely attributable to an auditory or linguistic framework established prior to deafness (Glick and Sharma 2017). These pre-existing neural networks may allow for more efficient cortical reorganization when auditory input is reintroduced via a CI (Kral and Sharma 2012).

While N2b amplitude trended higher in postlingual CI users, the most pronounced effect emerged in the P3a component, which is associated with attention and novelty detection. The significant correlation between P3a amplitude and age at deafness onset emphasizes the role of prior auditory exposure in shaping higher-order cognitive processes (Eggermont and Ponton 2003; Nicholas and Geers 2006). These findings align with research showing that later-latency components, such as P3a, are particularly sensitive to experiential differences in auditory exposure and language acquisition (Sharma and Dorman 2006). They are also consistent with recent evidence from children with CIs showing that reduced P3 amplitude is associated with poorer language and literacy outcomes, further underscoring the sensitivity of these components to individual differences in auditory experience (Deroche et al. 2023).

Interestingly, such effects were not observed when stratifying the CI group by age at implantation or duration of deafness. This suggests that the age at onset of deafness exerts a strong influence on higher-order processes, as it determines whether critical auditory and linguistic networks were established during sensitive periods (Lenneberg 1967). However, the contributions of other factors, such as duration of auditory deprivation before implantation and auditory experience postimplantation, cannot be excluded and warrant further investigation in larger, more balanced cohorts (Dettman et al. 2007).

These observations support sensitive-period frameworks (Knudsen 2004), which propose that early auditory deprivation differentially affects the maturation of language and attentional networks (Kral and Tillein 2006). Individuals who experienced auditory input during the sensitive period may retain neural pathways that facilitate faster processing of auditory information (Glick and Sharma 2017). Conversely, prelingually deafened individuals often lack these templates, leading to slower and less efficient engagement of higher-order processes.

Future research should examine how early and sustained auditory input, including early cochlear implantation, alters these patterns. Longitudinal designs could reveal how ERP differences evolve with prolonged CI use and auditory experience, shedding light on the long-term trajectory of cortical reorganization in pre- and postlingually deafened individuals.

### Implications for rehabilitation and speech perception in CI users and blind individuals

Our findings reveal important distinctions in how sensory-deprived populations process speech. While CI users exhibited deficits in P2 and N2b amplitudes and prolonged latencies, blind individuals demonstrated enhanced auditory processing capabilities, including faster MMN latencies and heightened P3a amplitudes. These findings highlight the need for personalized rehabilitation strategies.

For prelingually deafened CI users, rehabilitation should prioritize strengthening attentional engagement to improve their ability to process auditory stimuli effectively, as measured by the P3a. Structured programs could include auditory discrimination tasks, phoneme categorization exercises, and real-world sound detection challenges to enhance cognitive processes, particularly in environments with competing auditory stimuli or subtle phonetic distinctions (Beynon and Snik 2004; Zhang et al. 2011). Additionally, interventions designed to foster attentional engagement and novelty detection, such as oddball paradigms or fast-paced auditory games, could improve P3a-related functions, enabling better adaptation to changing auditory environments.

For post-lingually deafened CI users, the residual linguistic framework established before deafness offer a foundation for targeted interventions. Rehabilitation for this group can focus on leveraging these networks to refine higher-order processes, such as attention-switching and auditory integration. These strategies align with research indicating that postlingual auditory experience allows for faster cortical reorganization and superior outcomes in higher-order auditory tasks (Glick and Sharma 2017).

It is important to note that these findings primarily apply to CI users implanted later in life, as our sample did not include individuals who received implants early. Early implantation is known to mitigate many of the deficits associated with prelingual deafness by preserving sensitive periods for auditory development (Sharma and Dorman 2006; Dettman et al. 2007). Early auditory stimulation via CI may facilitate stronger outcomes in both early sensory processing and higher-order cognitive functions. Future studies should investigate how early implantation influences ERP components like N2b and P3a to refine our understanding of optimal intervention timing.

For blind individuals, understanding their compensatory mechanisms—such as faster ERP latencies and enhanced P3a amplitudes—can inform the design of training programs to improve auditory capabilities. For example, auditory-based navigation tools or speech-processing systems that exploit their superior temporal resolution and attentional engagement could improve communicative efficacy (Merabet and Pascual-Leone 2010). These approaches could also be integrated into broader rehabilitation programs for individuals with combined sensory impairments.

## Conclusion

This study highlights how auditory and visual deprivation differentially affect speech perception and cortical processing, as reflected in ERP components. Blind individuals demonstrated faster and more efficient auditory processing, likely driven by cross-modal plasticity, while CI users—particularly prelingual CI users—faced challenges in both early sensory processing and higher-order cognitive control. These findings emphasize the necessity of adapting rehabilitation strategies to align with the individual’s auditory and linguistic history in cases of auditory deprivation while leveraging the auditory strengths of blind individuals to enhance rehabilitation outcomes.

Future research should investigate the long-term effects of early and sustained auditory input on cortical reorganization and speech perception outcomes, especially in prelingually deafened individuals. This work also emphasizes the broader implications of cross-modal plasticity and cognitive adaptation for designing innovative, individualized sensory rehabilitation strategies that improve communicative efficacy and quality of life.

## Data Availability

Data are available upon reasonable request.
